# The Nuclear Hormone Receptor PPAR_γ_ as a Therapeutic Target in Major Diseases

**DOI:** 10.1100/tsw.2010.213

**Published:** 2010-11-04

**Authors:** Martina Victoria Schmidt, Bernhard Brüne, Andreas von Knethen

**Affiliations:** Institute of Biochemistry I-Pathobiochemistry, Faculty of Medicine, Goethe-University Frankfurt, Germany

**Keywords:** PPAR_γ_, immune regulation, sepsis, atherosclerosis, cancer

## Abstract

The peroxisome proliferator-activated receptor γ (PPAR_γ_) belongs to the nuclear hormone receptor superfamily and regulates gene expression upon heterodimerization with the retinoid X receptor by ligating to peroxisome proliferator response elements (PPREs) in the promoter region of target genes. Originally, PPAR_γ_ was identified as being essential for glucose metabolism. Thus, synthetic PPAR_γ_ agonists, the thiazolidinediones (TZDs), are used in type 2 diabetes therapy as insulin sensitizers. More recent evidence implied an important role for the nuclear hormone receptor PPAR_γ_ in controlling various diseases based on its anti-inflammatory, cell cycle arresting, and proapoptotic properties. In this regard, expression of PPAR_γ_ is not restricted to adipocytes, but is also found in immune cells, such as B and T lymphocytes, monocytes, macrophages, dendritic cells, and granulocytes. The expression of PPAR_γ_ in lymphoid organs and its modulation of macrophage inflammatory responses, lymphocyte proliferation, cytokine production, and apoptosis underscore its immune regulating functions. Moreover, PPAR_γ_ expression is found in tumor cells, where its activation facilitates antitumorigenic actions. This review provides an overview about the role of PPAR_γ_ as a possible therapeutic target approaching major, severe diseases, such as sepsis, cancer, and atherosclerosis.

## PPARγ-DEPENDENT ANTI-INFLAMMATORY MECHANISMS

Inflammatory conditions typify the host's response to external challenges, or cellular injury causes activation of diverse inflammatory mediators to modify tissue structure and function. A prolonged inflammatory state with the release of proinflammatory cytokines and reactive oxygen species (ROS) from activated leukocytes can, in turn, destruct tissue, thereby contributing to the pathogenesis of many disease states[1,2]. Therefore, attention is being paid to factors that operate as negative regulators of inflammatory responses. Recently, peroxisome proliferator-activated receptor γ (PPARγ; NR1C3), a member of the nuclear receptor superfamily, has been demonstrated to provoke and mediate anti-inflammatory signaling[3]. Originally, it was identified as a key regulator of adipocyte differentiation[4] and glucose metabolism[5]. PPARγ acts as a transcriptional activator of many adipocyte-specific genes involved in lipid synthesis, handling and storage of lipids, growth regulation, insulin signaling, and adipokine production[6]. PPARγ binds to peroxisome proliferator response elements (PPREs) as a heterodimer with members of the retinoid X receptor (RXR; NR2B) subfamily, provoking target gene expression. Moreover, PPARγ has been shown to inhibit the expression of proinflammatory cytokines, such as tumor necrosis factor α (TNFα), interleukin (IL)-1β, and IL-6[2,7]. In monocytes/macrophages, PPARγ activation suppresses inducible nitric oxide synthase (iNOS) up-regulation[8] and ROS production[8,9]. Identification of molecular mechanisms responsible for the anti-inflammatory action of PPARγ is thus likely to be of practical importance in the efforts to develop safer and more effective drugs for the treatment of diseases associated with or caused by chronic or overwhelming inflammation.

So far, five different mechanisms are proposed for PPARγ to alter gene expression (Fig. 1)[2,10]. A major mechanism, mediated by the ability of PPARγ to cross-talk with proinflammatory signaling pathways, interfering with transcription factor activation has been termed transrepression. Transrepression involves protein-protein interactions between PPARγ and transcription factors, such as NF-κB[11,12,13], NF-AT[14], AP-1[13,15], or STAT[16] (Fig. 1A). This mechanism scavenges transcription factors from binding to their responsive elements rather than a direct sequence-specific interaction of PPARγ with DNA itself. This assigns PPARγ the ability to inhibit proinflammatory signaling and to induce an anti-inflammatory response. In immune cells, PPARγ activation thus attenuates expression of proinflammatory mediators, such as IL-2, TNFα, IL-1β, and IL-12. PPARγ-dependent transrepression can also be mediated by PPARγ binding to coactivator complexes, such as steroid receptor co-activator 1 (SRC1) and cAMP response element binding (CREB)-binding protein (CBP)/p300, which are essential for transcription factor–dependent transactivation (Fig. 1B). These coactivator complexes usually reorganize the chromatin packaging through their histone acetyltransferase activity, to allow the transcriptional machinery gaining access to the promoter region[17]. Because these transcriptional coactivators are indispensable for AP1-mediated gene induction[18] and enhance NF-κB–dependent transcription[19], proinflammatory gene expression is inhibited. SUMOylated PPARγ can transrepress gene expression by preventing removal of the corepressor complex nuclear receptor corepressor/silencing mediator for retinoid and thyroid hormone receptors (NCoR/SMRT) from the promoter region of proinflammatory transcription factors, e.g., NF-κB, which, through association with histone deacetylases (HDACs), attenuates gene transcription (Fig. 1C)[20,21]. Besides transrepression, PPARγ can inhibit mitogen-activated protein kinase (MAPK) by a so-far-unknown mechanism[22,23,24]. This inhibition prevents MAPK from phosphorylating and thereby activating downstream transcription factors, which are necessary for MAPK-dependent proinflammatory gene expression (Fig. 1D). Additionally, cytosolic PPARγ is able to bind protein kinase Cα (PKCα) directly, subsequently inhibiting PKCα translocation to the cell membrane, its activation, and further depletion. This provokes monocyte/macrophage desensitization in response to apoptotic cell phagocytosis, where attenuated PKCα signaling blocks NADPH-oxidase–dependent ROS formation (Fig. 1E)[25]. Casein kinase II (CK-II)–dependent PPARγ phosphorylation makes PPARγ accessible for the CRM1-dependent nuclear export machinery, consequently triggering cytosolic PPARγ localization. This process involves RanGTP and phosphorylation of RanBP3 to shuttle PPARγ to the cytosol efficiently[9,26].

## CONSEQUENCES OF PPARγ ACTIVATION IN IMMUNE CELLS

PPARγ activation in immune cells predominantly results in transrepression of proinflammatory gene expression. PPARγ agonists have been shown to decrease IL-2 production in activated T cells and thereby to enhance apoptosis[14,27,28,29]. Additionally, PPARγ ligands can inhibit activation-induced production of several T-cell cytokines, including the classical $T_H$1-cell cytokine interferon γ (IFNγ)[30]. The ability of ligand-activated PPARγ to inhibit IL-12 production by dendritic cells, as well as its ability to inhibit IFNγ production by T cells, indicates that this nuclear hormone receptor might play a role during differentiation of naïve T cells into their effector subsets. Conversely, it was shown that the presence

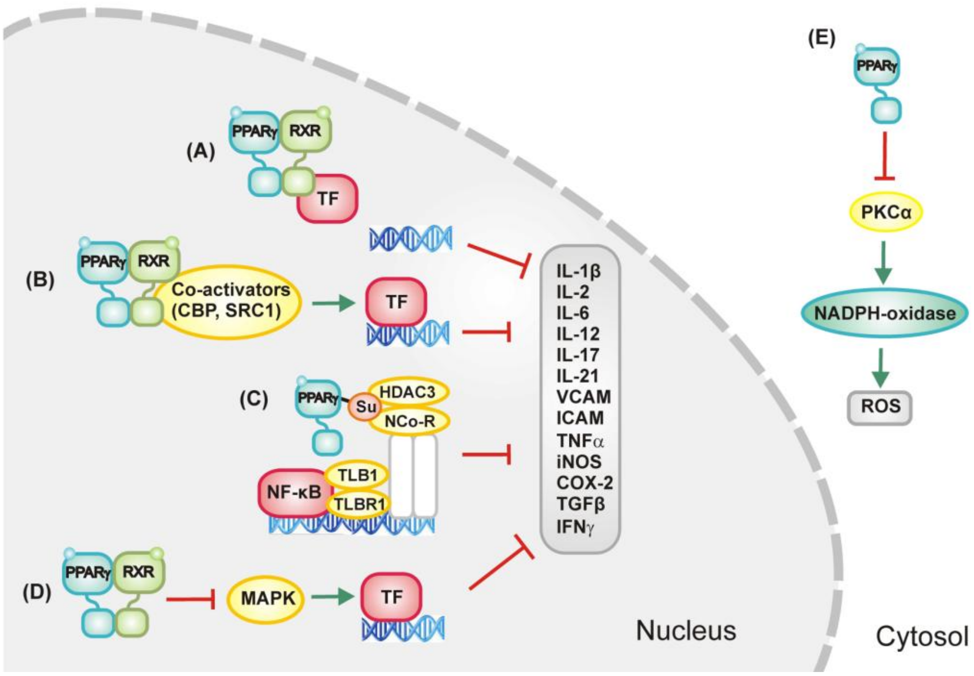

**FIGURE 1.** Mechanisms of PPARγ-dependent inhibition of proinflammatory gene expression. In response to agonist, PPARγ can form a heterodimer with the RXRα. In this heterodimeric state, PPARγ (A) directly associates with proinflammatory transcription factors (TF), such as NF-κB, STAT, and AP-1, and prevents proinflammatory gene expression; (B) scavenges transcriptional coactivators, such as CBP and SRC-1 from binding to proinflammatory transcription factors (TF), enhancing the expression of proinflammatory genes. (C) In response to its SUMOylation (Su), PPARγ inhibits proteasomal degradation of the NCoR/HDAC3/TLB1/TLBR1 complex, which in turn would allow NF-κB–dependent proinflammatory gene expression. (D) Besides these three mechanisms of transrepression, PPARγ inhibits MAPK to attenuate downstream signaling, which is necessary to induce proinflammatory gene expression. (E) Finally, activated PPARγ is assumed to inhibit PKCα translocation, thereby attenuating NADPH oxidase activation. In C and E, heterodimer formation with RXRα is not required.

of IL-4, a cytokine important for the development of $T_H2$ cells[31], can provide a potential PPARγ-specific ligand through up-regulation of the 12/15 lipoxygenase in monocytes[32]. This enzyme produces 13-hydroxyoctadecadienoic acid (13-HODE), which in turn suppresses the production of IL-4 in monocytes. Furthermore, 13-HODE can be taken up by T cells to activate PPARγ, thus promoting a $T_H2$ effector phenotype, where PPARγ is highly expressed[32,33]. These observations suggest that PPARγ may contribute to the anti-inflammatory activities of IL-4 and that full proinflammatory activities require down-regulation of PPARγ. Previous results demonstrated that the inhibitory effects of the PPARγ agonist rosiglitazone upon LPS and IFNγ stimulation are PPARγ dependent when the drug is used at concentrations close to its binding affinity. At high concentrations, the inhibitory effects are PPARγ independent and probably mediated, at least in part, by the unspecific activation of PPARδ[34].

Further, PPARγ plays a crucial role in autoimmune diseases, such as multiple sclerosis. Th17 cells provide protection towards (certain) infections, but more importantly, these cells have been linked to the development of autoimmunity. Th17 differentiation critically depends on transforming growth factor β (TGFβ), together with proinflammatory cytokines like IL-6 or IL-21. Thus, PPARγ blocks TGFβ/IL-6–dependent expression of the retinoid receptor–related orphan receptor γt (RORγt), the key transcription factor for Th17 differentiation, making them more amenable to pharmacological modulations[35].

## PPARγ AND SEPSIS

In the course of sepsis, one of the most severe inflammatory processes, PPARγ acts as a double-edged sword. On the one hand, by attenuating NF-κB transactivation, PPARγ inhibits cytokine expression of, for example, TNFα or IL-12[36], the expression of vascular cell adhesion molecule-1 (VCAM-1), and intercellular adhesion molecule-1 (ICAM-1)[37]. Moreover, the expression of the inducible proinflammatory proteins, cyclo-oxygenase-2[15], cytosolic phopholipase A2, and inducible nitric oxide synthase (iNOS)[8], are blocked[38,39]. *In vivo* studies showed that administration of the PPARγ agonist 15-deoxy-$\Delta^{12,14}$-prostaglandin J2 (15d-$PGJ_2$) prior to polymicrobial sepsis reduces lung injury and neutrophil trafficking to the lung and small intestine[40]. 15d-$PGJ_2$ enhances PPARγ functions in the lung, decreases NF-κB activity, and promotes expression of the cytoprotective heat shock protein 70 (HSP70)[41]. In turn, the heat shock response is amplified and correlates well with improved lung injury. In the study of Matsui et al., it is proposed that the ability of 15d-$PGJ_2$ to block cytokine-induced iNOS in macrophages is associated with the expression of HSP70[42]. Correspondingly, the duration and extent of NF-κB activation corresponds with the severity of the inflammatory response, i.e., it persists longer in nonsurviving than in surviving patients with acute sepsis. In these studies, it has been suggested that PPARγ modulates expression of inflammatory genes by transrepression. However, 15d-$PGJ_2$ has also been reported to alter NF-κB activation directly, independent of PPARγ in sepsis[43]. Straus et al. provided mechanistic evidence that 15d-$PGJ_2$ directly inhibits NF-κB–dependent gene expression through covalent modifications of critical cysteine residues in IκB kinase and the DNA-binding domains of NF-κB subunits[44].

Conversely, PPARγ seems to play a pivotal role in the compensatory anti-inflammatory response syndrome. This describes the later phase in the course of sepsis, where the body responds with anti-inflammatory cytokine production as well as a massive apoptosis of immune cells to diminish self-harmful effects[45,46]. Hotchkiss and colleagues even showed that depletion of lymphocytes is the central pathogenic event during sepsis and contributes severely to a bad outcome of sepsis[45,47,48,49,50]. In mice, blocking T-cell apoptosis by overexpression of the antiapoptotic Bcl-2 protein[51,52,53] or by depletion of proapoptotic Bim[54,55,56,57,58] resulted in a higher survival rate compared to wild-type controls in response to the cecal ligation and puncture (CLP)–dependent polymicrobial sepsis. Previous results of our group showed that PPARγ also induces apoptosis in T cells by a yet-unknown mechanism. When primary human T cells of septic patients are stimulated with PPARγ agonists, they undergo apoptosis. In line, in these activated T cells, PPARγ expression is increased. Moreover, the serum of sepsis patients provoked PPARγ transactivation, suggesting that it contains a specific PPARγ agonist whose nature has so far not been clarified[59,60]. PPARγ activation during the onset of sepsis, as an endogenous anti-inflammatory factor, is suggested to prevent sepsis progression by attenuating the hyperinflammatory response. During the later phase of sepsis, PPARγ activation might enhance immune paralysis by contributing to T-cell apoptosis, thus worsening the septic outcome. Therefore, under these conditions, antagonizing PPARγ might be more appropriate.

## PPARγ AND CANCER

In tumorigenesis, it has been controversially discussed whether the role of PPARγ is pro-oncogenic or antineoplastic. Previous observations suggest a multifaceted and tissue-specific effect of PPARγ. For colon cancer, it has been shown that PPARγ retards the growth of cultured cell carcinomas or implanted tumors, whereas in colon epithelium, proliferation is favored. Tumor-restricted PPARγ expression, as shown for colon, breast, and bladder cancer, modifies tumor development, prolonging survival, lowering mortality, and consequently accounting for a good prognosis. Indeed, more and more studies demonstrate a number of antineoplastic processes initiated by PPARγ. In this line especially, its antiproliferative effects, such as arresting the cell cycle and causing cell differentiation, as well as inhibition of angiogenesis and induction of apoptosis, are discussed as possible antineoplastic mechanisms of PPARγ.

In 2001, Patel et al. observed that PPARγ induces expression of the phosphatase and tensin homolog on chromosome ten (PTEN) in MCF-7 breast carcinoma and $CaCo_2$ colon cancer cell lines, possibly by binding to two putative PPREs identified within the PTEN promoter[61]. As a result of up-regulated PTEN, Akt phosphorylation and concomitant cell proliferation was significantly reduced. Two other studies further confirmed this initial report[62,63]. Moreover, in the latter, it was shown that PPARγ-dependent PTEN expression can be blocked by estrogen receptor binding to the PPRE site of the PTEN promoter[63]. This cross-talk has to be carefully considered when new therapy approaches are developed.

Through the identification of genetically damaged cells, the tumor-suppressor protein p53 minimizes the risk of harmful mutations by either pausing the cell cycle for repair or eliminating the cell by apoptosis. p53 achieves many of its roles through induction of target genes, to arrest the cell cycle via p21(Cip1/WAF1) or to induce apoptosis via the proapoptotic Bax. Of particular significance in this line is the ability of PPARγ to enhance p53 expression, by binding to the NF-κB–responsive element, located in the promoter region of p53[64]. However, in 2008, Zaytseva et al. demonstrated that down-regulation of PPARγ suppresses cell growth and induces apoptosis in MCF-7 breast cancer cells[65]. Previous work showed that a distinct promoter regulates PPARγ expression in MCF-7 cells, and that promoter switching causes differential PPARγ expression between normal and cancer cells[66,67]. Recently, the authors provided evidence that a knockdown of PPARγ in MCF-7 cells blocks proliferation, decreasing cell transition from the G1 to the S phase. Since these studies were performed only in breast cancer cell lines, one may speculate that these observations are cell-type specific. PPARγ has been shown to alter carcinogenesis in other tumors as well. A recent work by Yu et al. implicated an inhibitory role of PPARγ in hepatocarcinogenesis[68]. In this case, an animal model was used to genetically ablate PPARγ expression on one allele ($PPAR\gamma^{+/-}$), which decreases PPARγ expression, but is not lethal during embryogenesis like total PPARγ knockout ($PPAR\gamma^{-/-}$). Using a diethylnitrosamine (DEN)-induced hepatocarcinoma cell (HCC) model, the authors showed that activation of PPARγ by rosiglitazone blocked tumor development in PPARγ wild-type ($PPAR\gamma^{+/+}$) littermates, whereas it did not alter tumor formation in $PPAR\gamma^{+/-}$ mice. To elucidate the underlying mechanism, the authors transduced the human hepatoma cell line Hep3B with a PPARγ-expressing adenovirus. In these transduced cells, PPARγ overexpression induced a G2/M arrest and apoptosis, mediated by extrinsic (Fas+TNFα) and intrinsic (caspases 3, 7, 9, and PARP) pathways. Both, cell cycle arrest and cell death were additively enhanced in response to rosiglitazone-mediated PPARγ activation. Moreover, PPARγ overexpression induced expression of the growth differentiation factor-15 (GDF-15), which has been established to attenuate proliferation in other tumor systems as well[69,70,71]. These *in vitro* data were finally corroborated in the liver tissue of $PPAR\gamma^{+/+}$ mice by immunohistochemistry. There, GDF-15 expression in response to rosiglitazone significantly increased compared to untreated control animals or rosiglitazone-stimulated heterozygous littermates. The authors conclude that PPARγ functions as a tumor suppressor in hepatocarcinogenesis[68].

In other studies, impairment of the intact PPARγ permits tumorigenesis. In a subset of thyroid follicular carcinomas, a chromosomal translocation (t(2;3)(q13;p25)) results in the expression of a paired box gene-8 (PAX8)-PPARγ fusion protein[72]. This fusion protein cannot be activated by agonists such as rosiglitazone and, thus, constitutes a dominant-negative protein. Overexpression of the PAX8/PPARγ fusion protein stimulated the proliferation of primary human thyroid cells, suggesting that the PPARγ moiety-dependent transactivation of this fusion protein is an essential component in its tumorigenic action[73,74]. For other cancers, such as bladder cancer, it was shown that mutations block the formation and functionality of PPARγ. Hence, heterodimerization with RXR and binding to the responsive element on the DNA is inhibited, impairing the induction of antitumorigenic activity.

Colon cancer cells that were exposed to PPARγ exhibited a G1 cell cycle arrest and increased expression of the carcinoembryonic antigen. Moreover, activation of PPARγ up-regulates cavedin-1, a tumor-suppressor protein. This was abrogated by treatment with the PPARγ antagonist GW9662, showing that PPARγ agonist–induced tumor suppression is specifically initiated by activation of the PPARγ pathway. This was confirmed in a mouse model of human bladder cancer, where treatment with PPARγ agonists significantly inhibited tumor growth[75].

In human glioma cells, stimulation with ciglitazone or troglitazone resulted in a concentration- and time-dependent apoptotic cell death. Herein, ciglitazone-induced ROS generation and cell death were prevented by the antioxidant N-acetylcysteine, suggesting an important role of ROS generation for ciglitazone-induced apoptosis. Apoptosis could be inhibited by the PPARγ antagonist GW9662[76]. Thus, induction of PPARγ-dependent apoptosis may be a promising therapeutic approach in cancer therapy.

Tumor development is associated with accumulation of genetic changes, which ultimately produce a pronounced inflammatory phenotype that is also aggressive in growth and relatively resistant to chemotherapy. Although acute inflammation is a necessary process aimed at protecting the organism after an injury, unresolved chronic inflammation may promote cancer formation by providing an appropriate environment for tumor growth[77,78,79]. Although mechanisms that link inflammation and cancer have only been studied rudimentarily, epidemiological studies show a convincing association between them[80,81]. Thus, cytokines that are released in response to infection, inflammation, and immunity can function to inhibit tumor development and progression. Alternatively, cancer cells can respond to host-derived cytokines that promote growth, attenuate apoptosis, and facilitate invasion and metastasis. Proinflammatory cytokines implicated in carcinogenesis include IL-1, IL-6, IL-15, colony stimulating factor, TNFα, and the macrophage migration inhibitory factor. In this context, tumor-associated macrophages (TAM) seem to be especially important. They display a phenotype similar to M2-polarized macrophages, which are characterized by the expression of anti-inflammatory cytokines such as IL-10 and proangiogenic factors, e.g., vascular endothelial growth factor (VEGF)[82]. By contributing to vascular supply and to vascular endothelial cell proliferation, they play a critical role in the maintenance and development of cancer. Fauconnet et al. showed that PPARγ expression correlates with VEGF expression. This was verified by reporter gene experiments showing ligand-mediated PPARγ-dependent VEGF transcription[83]. Macrophage polarization to the M2 phenotype has been linked in part to PPARγ activation[84,85,86], mainly attributed to its role in transrepressing proinflammatory gene expression. Recently, in a mouse model of progressing T lymphoma, M2 macrophages blocked cytotoxicity of $CD8^+$ T lymphocytes (CTL)[87]. Stimulation of these M2 macrophages with PPARγ agonists restored CTL-dependent cytotoxicity towards tumor cells. Therefore, PPARγ might be a new potential therapeutic target to abrogate macrophage-mediated CTL suppression in cancer. Additional support for a role of PPARγ in macrophage-dependent attenuation of tumor development came from a study by Shah et al. The authors provide evidence that mice with a targeted disruption of PPARγ in macrophages displayed an increased susceptibility to dextran sulfate sodium (DSS)–induced colitis compared to PPARγ wild-type littermates[88]. The data suggest that PPARγ regulates macrophage recruitment to inflammatory foci in the colon.

The role of PPARγ in physiology and pathophysiology should be clarified, since this may increase our understanding of how cancer occurs and how it can be treated. While the identities of target genes that contribute to the antiproliferative activities of PPARγ agonists remain largely elusive, accumulating evidence indicates that TZDs mediate PPARγ-independent antitumor effects by targeting diverse signaling cascades governing proliferation and survival of cancer cells[89]. Several studies using PPARγ-deficient mouse models support this assumption. PPARγ deletion in hepatocytes in a hepatitis B virus transgenic mouse model did not alter hepatic carcinogenesis, but increased the TZD-mediated antitumorigenic effect when compared to PPARγ wild-type mice[90]. Moreover, TZDs inhibited invasiveness of pancreatic cancer cells derived from human pancreatic adenocarcinomas via PPARγ-independent mechanisms[91]. Although TZDs can attenuate tumor development or progression PPARγ independently, many human cancer cells have been shown to exhibit high levels of PPARγ. Exposure of these tumor cells to high doses of PPARγ agonists provoked cell cycle arrest, apoptosis, and redifferentiation, suggesting a link between PPARγ signaling and the antitumor activities of PPARγ ligands. Energy balance, obesity, and lack of exercise are leading nongenetic predispositions that cause an increased tumor risk. PPARγ could herein act as the potential link between energy balance, cellular metabolism, and cancer pathogenesis. However, the exact role of PPARγ during carcinogenesis and tumor cell growth is still unclear.

## PPARγ AND ATHEROSCLEROSIS

Activation of PPARγ is an important anti-inflammatory strategy during inflammation[17]. Originally, PPARγ was described as a transcription factor belonging to the hormone receptor superfamiliy, localized in the nuclear membrane, regulating glucose and lipid metabolism[5]. Thus, a major role of PPARγ in the pathogenesis of obesity, diabetes, and arteriosclerosis can be deviated[92]. The antidiabetic synthetic TZDs operate by specifically activating PPARγ[93,94]. These drugs enhance the effect of insulin and lower the level of serum glucose[95]. A recent review by Kaul et al. summarizes evidence from recent trials analyzing the long-term effect of TZD use. These data suggest that rosiglitazone is associated with an increased risk of cardiovascular events, and pioglitazone and rosiglitazone are associated with higher rates of heart failure[96]. PPARγ activation by the TZDs results in an array of effects on traditional and nontraditional cardiovascular risk factors that are independent of their effects on glycemic control. These include, for example, reduction of intermedia thickness progression[97] and circulating platelet activity[98], attenuation of PAI-1 expression[99], inhibition of glycation[100], increase in plasma adiponectin[101], and reduction of CRP[102], IL-6[103], and MMP-9 levels[104]. However, further trials are necessary to determine to which extent the different factors contribute to TZD-dependent cardiovascular alterations.

During atherogenesis, PPARγ is expressed by activated monocytes and macrophages. As a first step, monocytes are attracted to the vessel wall of large arteries by activated endothelial cells. Having attached by binding to selectins and integrins, monocytes infiltrate into the subendothelial space, mostly following a chemokine gradient, e.g., IL-8, originating from the source of infection, where they differentiate to macrophages[105]. Already this step is altered in response to PPARγ activation. Thus, troglitazone inhibited formation of early atherosclerotic lesions in low-density lipid receptor knockout (LDL-R$^{-/-}$) mice by decreasing macrophage accumulation in the intima, consistent with the *in vitro* observation that rosiglitazone as well as troglitazone inhibited MCP-1–directed transendothelial migration of monocytes[106]. A study transplanting bone marrow derived from macrophage-specific PPARγ knockout (MΦ-PPARγ$^{-/-}$) mice into LDL-R$^{-/-}$ and control mice supported this observation. Macrophages derived from MΦ-PPARγ$^{-/-}$ showed increased migration and CC chemokine receptor 2 (CCR2) expression[107], which is the receptor for MCP-1. In the intima, macrophages scavenge trapped lipoproteins, such as oxLDL, leading to the formation of so-called foam cells and subsequent activation of these lipid-laden macrophages[108]. This contributes to the established designation of arteriosclerosis as a chronic inflammatory disease[109]. However, the role of PPARγ expression in these cells of monocytic origin is controversially discussed. Activation of PPARγ enhances oxLDL uptake by up-regulating CD36 expression, a class B scavenger receptor, consequently fostering foam cell formation[110]. On the other hand, PPARγ-dependent expression of the liver X receptor α (LXRα) provokes a decrease of intracellular cholesterol accumulation. As a heterodimer in combination with the RXR, LXRα induces expression of the ATP-binding cassette transporter A1 (ABCA1), causing lipid export from macrophages and thus counteracting foam cell generation[111]. Experiments using either PPARγ-negative embryonic stem cells or macrophages from MΦ-PPARγ$^{-/-}$ mice support both of the described PPARγ-dependent effects[112,113].

As recently reviewed by Mosser and Edwards[114], these differences might be attributed to macrophage plasticity. Macrophages are versatile immune cells that, depending on environmental stimuli, acquire diverse functional states. The extremes of this continuum are defined as the classically activated M1 and the alternatively activated M2 phenotype[115]. However, the alternative M2 definition does not satisfy and mirror the compelling evidence gathered over the last few years any more, since the M2 designation comprises macrophages with dramatic differences in their biochemistry and physiology[116].

The M1 macrophage phenotype is established in response to stimuli, like the bacterial cell wall component LPS and IFNγ, released mainly by $T_H1$ cells. These activators are rapidly generated following infection or injury. M1 macrophages are characterized by an enhanced microbicidal capacity, elicited by proinflammatory mediator production such as TNFα, IL-1β, IL-6, IL-12, NO, or ROS[117,118]. Concerning the stimulation regime provoking M1 macrophage activation, one has to keep in mind that LPS treatment rapidly down-regulates PPARγ after 6–15 h by miR27b-dependent mRNA destabilization[119], followed by its up-regulation again at later time points[120,121]. Support for this assumption came from Dos Santos Mendes et al., recently demonstrating that LPS stimulation of RAW 264.7 macrophages for 24 h transactivated PPARγ, as observed by PPRE reporter analysis[122]. In analogy, we have shown PPARγ DNA binding (EMSA) and transactivation (reporter assay) in response to LPS/IFNγ-treatment for 15 h, further underlining the notion of PPARγ activation after prolonged LPS stimulation[123].

Alternative activation of macrophages with, for example, glucocorticoids, IL-4, IL-13, or IL-10, induces an anti-inflammatory M2 phenotype, which is characterized by attenuated production of proinflammatory mediators, but enhanced secretion of anti-inflammatory mediators, such as IL-10, TGF-β1, and prostaglandin $E_2$ ($PGE_2$). The M2-type designation regroups several populations of macrophages with different biochemistry and function, like wound-healing (M2a), hybrid (M2b), and regulatory (M2c) macrophages[114,124]. Concerning these different M2 macrophage subpopulations, PPARγ activation primes monocytes for differentiation into alternative M2 macrophages with anti-inflammatory properties[84]. In their study Bouhlel et al. demonstrated that in human atherosclerotic lesions, the expression of M2 markers (AMAC-1, CD36, CD206, IL-10) and PPARγ positively correlated. Interestingly, PPARγ activation in resting primary human monocytes did not induce the M2 phenotype, whereas PPARγ agonists being applied in combination with IL-4/IL-13 enhanced expression of M2 markers compared to controls treated only with IL-4/IL-13. In addition, M2 macrophages retain a high level of PPARγ expression[85]. Because macrophages are plastic cells, they can switch from an activated M1 phenotype to M2, and vice versa, upon specific signals[125]. In 2000, Li et al. provided evidence that the PPARγ agonists rosiglitazone and GW7845 strongly inhibited development of atherosclerosis in male LDL-$R^{-/-}$ mice[126]. The antiatherogenic effect in male mice correlated with impaired insulin sensitivity and decreased tissue expression of TNFα. Using the apolipoprotein E knockout ($ApoE^{-/-}$) mouse model, Chen et al. demonstrated a similar outcome in response to the TZD rosiglitazone, attenuating fatty streak formation and accordingly inhibiting atherosclerosis[127]. Studies elucidating the role of PPARγ in advanced atherosclerotic lesions in LDL-$R^{-/-}$ mice found that TZDs cannot reverse, but block, atherosclerosis progression[128] or sometimes even enhance atherosclerosis by increasing macrophage apoptosis and plaque necrosis[129]. A gene therapy approach by a recombinant adenovirus carrying mouse PPARγ cDNA provoking overexpression of PPARγ in $ApoE^{-/-}$ mice already suffering from atherosclerosis attenuated atherosclerotic lesions and stabilized atherosclerotic plaques[130]. This study suggests that PPARγ might offer a promising gene therapeutic target, improving atherosclerosis formation.

Taken together, the role of PPARγ in atherosclerosis seems mainly atheroprotective. Therefore, therapeutic approaches might focus on prolonged PPARγ activation or intervention with PPARγ down-regulation. However, one should keep in mind that a main reason for atherosclerosis progression is the formation of the necrotic core in the intima, whose onset might be, at least in part, triggered by desensitized macrophages that are unable to remove the accumulating cellular debris completely[131,132]. Under these circumstances, activation of PPARγ, enhancing an anti-inflammatory macrophages phenotype, might be even more deleterious. Therefore, further research is necessary to elucidate fully the time response of a pro- vs. antiatherosclerotic role of PPARγ during atherosclerosis progression.

Apart from the three mentioned diseases, PPARγ is expressed in many other tissues/cells and, thus, is a possible therapeutic target in regulating other diseases. In part, preclinical studies using TZDs have been effective in several animal disease models. A brief overview is provided in Table 1.

**TABLE 1**
**Effects of TZD Treatments and PPARγ Gene Dosage on Rodent Models of Inflammation**

| Disease | Model | Intervention | Effect | Refs. |
|---|---|---|---|---|
| Experimental autoimmune encephalomyelitis | | Wild type vs. PPARγ$^{+/-}$ | ↑ Severity in PPAR γ$^{+/-}$ mice | [133] |
| | | PI, RO, GW | ↓ Incidence and severity in monophasic disease | [134,135] |
| | | TR | ↓ Severity | [136] |
| Colitis | DSS | TR, RO | ↓ Colonic inflammation | [137] |
| | TNBS | Wild type vs. PPARγ$^{+/-}$ | ↑ Colonic inflammation in PPAR γ$^{+/-}$ mice | [24] |
| | | TR, RO | ↓ Colonic inflammation | [24,138] |
| | DSS/azoxymethane | TR | ↓ Colonic inflammation | [139] |
| | DSS | Wild type vs. PPARγ$^{+/-}$ | ↑ Colonic inflammation and mortality in PPAR γ$^{+/-}$ mice | [140] |
| | | TR, RO, PI | ↓ Colonic inflammation (only before disease initiation) | |
| Arthritis | CIA | RO | ↓ Incidence and severity | [141] |
| | AIA-CFA | Wild type vs. PPARγ$^{+/-}$ | ↑ Severity in PPAR γ$^{+/-}$ mice | [142] |
| | AIA-CFA | PI, RO | ↓ Inflammation | [143] |
| Obesity | ob/ob | Diet-induced obesity | ↑ Adipose tissue inflammation | [144] |
| | | RO | ↓ Macrophage infiltration; ↓ adipose tissue inflammation | |
| Airway inflammation | TDI-induced asthma | Ad-PPARγ, RO, PI | ↓ Symptoms of asthma | [145] |
| | Murine asthma | CI, RO, PG, GI, SB | ↓ Eosinphilic influx, mucus production, serum IgE | [146] |
| | LPS-induced neutrophilia | | ↓ Neutrophila | |
| | Pulmonary fibrosis | | ↓ Edema, lung injury | |
| Pancreatitis | Cerulein-induced pancreatitis | RO | ↓ Pancreatic inflammation, neutrophil inflitration | [147] |
| Psoriasis | Epidermal hyperplasia | CI, TR | ↓ Epidermal keratinocyte proliferation; ↑ differentiation | [148] |
| Acute inflammation | Carrageenan-induced edema or pleurisy | RO | ↓ Paw edema, pleural exudate formation, mononuclear cell infiltration, histological injury | [149] |

Abbreviations: Ad, adenovirus; AIA, adjuvant-induced arthritis; CFA, complete Freund´s adjuvant; CI, ciglitazone; CIA, collagen-induced arthritis; DSS, dextran sulfate sodium; GW, GW7845 ; ob/ob, leptin-deficient mice; PG, 15d-$PGJ_2$; PI, pioglitazone; Ro, rosiglitazone; SB, SB219994; TDI, toluene diisocyanate; TNBS, 2,4,6-trinitrobenzene sulfonic acid; TR, troglitazone.

## CONCLUDING REMARKS

It is hypothesized that the anti-inflammatory action of PPARs may explain the protective effects of these receptors in sepsis, atherosclerosis, cancer, and other inflammatory diseases (Fig. 2). However, emerging evidence for proinflammatory activities of activated PPARs is arising in light of new studies that associate PPARγ modulators with an increased incidence of both cardiovascular events in humans and sporadic formation of tumors in rodents. The antidiabetic TZDs are drugs that signal mainly via PPARγ and are used in clinical practice. A series of mechanistic studies revealed that TZDs, at clinically relevant concentrations, also bind and activate the transcriptional activity of PPARδ[34]. Collectively, these studies suggest that the observed proinflammatory and potentially deleterious effect of PPARγ ligands may

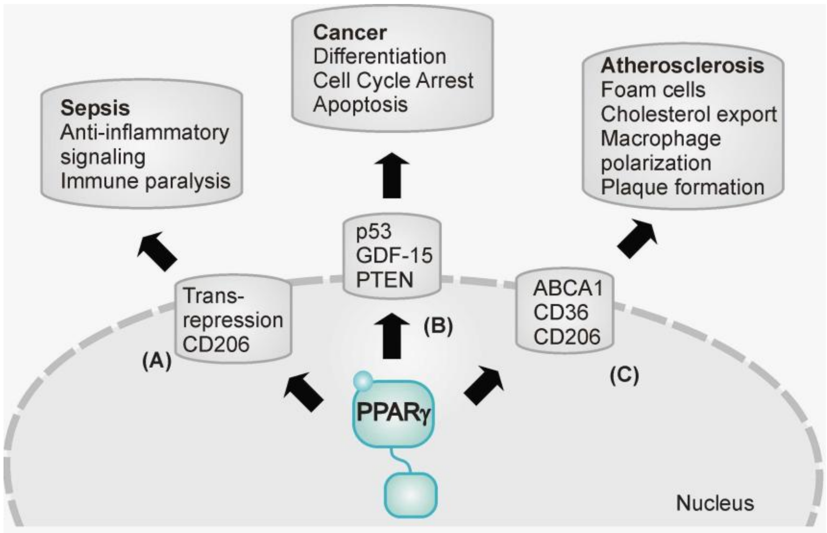

**FIGURE 2.** Summary of PPARγ-dependent effects in sepsis, cancer, and atherosclerosis. (A) In sepsis, the role of PPARγ is controversial. Transrepression of proinflammatory signaling during the initial phase of sepsis attenuates overwhelming inflammation, but may provoke insufficient bacterial clearance. Immune paralysis characterized by monocyte/macrophage desensitization as well as lymphocyte apoptosis is caused, in part, by PPARγ. Thus, PPARγ contributes to M2 macrophage polarization, for example, by induction of the M2 marker CD206 and repression of proinflammatory cytokine expression. Moreover, PPARγ seems to play an important role in lymphocyte apoptosis by a so-far-unidentified mechanism. (B) Cancer initiation and progression are inhibited by PPARγ-mediated expression of the tumor suppressor p53, the antiproliferative operating phosphatase PTEN, and the cell cycle arresting GDF-15. (C) PPARγ activation is involved in atherosclerosis by inducing CD36 expression, increasing oxLDL uptake, consequently fostering foam cell formation and contributing to M2 macrophage polarization. This might be involved in plaque formation due to incomplete phagocytosis of apoptotic debris and concomitant generation of the necrotic core. However, PPARγ-dependent induction of ATP-binding cassette transporters, such as ABCA1, provokes enhanced cholesterol export, thus counteracting atherosclerosis progression.

be mediated through an off-target effect on PPARδ. These studies highlight the need for PPAR modulators with increased receptor subtype specificity. Furthermore, they suggest that differences in systemic exposure and, consequently, in the activation of PPARγ and PPARδ may explain why TZDs can exhibit both inflammatory and anti-inflammatory activities in humans[150]. Although their supposed carcinogenic or toxic effects have to be monitored carefully, PPARs are important therapeutic targets. Many valuable approaches are now under consideration. Cell type– or tissue-specific PPARγ knockout mice, or humanized mice carrying the human PPARγ gene, will help to improve the understanding of the various actions of PPARγ in major diseases. In addition, the development of selective PPAR modulators (SPPARMs), rather than PPAR full agonists, which retain most of the benefits while reducing the adverse effects of PPAR activation, is a promising approach. Moreover, antagonizing PPARγ might also be of therapeutic importance. For example, this might improve sepsis outcome by attenuating T-cell depletion, thus preventing immune paralysis. Dependent on the respective tumor type, PPARγ activation or antagonization might block tumor development and progression. Finally, there is a similar picture in atherosclerosis. In the initial phase, PPARγ activation might prevent inflammatory conditions in the vessel and/or intima, which will significantly delay/retard plaque formation. At a later point of time, antagonizing PPARγ might be more attractive in order to avoid deficient removal of apoptotic debris. For all these reasons, PPARγ is certainly a useful therapeutic target to be explored further in the context of major diseases, such as sepsis, cancer, and atherosclerosis[79].

## References

1. Abdelrahman, M., Sivarajah, A., and Thiemermann, C. (2005) Beneficial effects of PPAR-gamma ligands in ischemia-reperfusion injury, inflammation and shock. *Cardiovasc. Res.* **65**, 772–781.
2. Pascual, G. and Glass, C.K. (2006) Nuclear receptors versus inflammation: mechanisms of transrepression. *Trends Endocrinol. Metab.* **17**, 321–327.
3. Straus, D.S. and Glass, C.K. (2007) Anti-inflammatory actions of PPAR ligands: new insights on cellular and molecular mechanisms. *Trends Immunol.* **28**, 551–558.
4. Farmer, S.R. (2006) Transcriptional control of adipocyte formation. *Cell Metab.* **4**, 263–273.
5. Willson, T.M., Lambert, M.H., and Kliewer, S.A. (2001) Peroxisome proliferator-activated receptor gamma and metabolic disease. *Annu. Rev. Biochem.* **70**, 341–367.
6. Lehrke, M. and Lazar, M.A. (2005) The many faces of PPARgamma. *Cell* **123**, 993–999.
7. Szeles, L., Torocsik, D., and Nagy, L. (2007) PPARgamma in immunity and inflammation: cell types and diseases. *Biochim. Biophys. Acta* **1771**, 1014–1030.
8. Li, M., Pascual, G., and Glass, C.K. (2000) Peroxisome proliferator-activated receptor gamma-dependent repression of the inducible nitric oxide synthase gene. *Mol. Cell. Biol.* **20**, 4699–4707.
9. von Knethen, A., Soller, M., Tzieply, N., Weigert, A., Johann, A.M., Jennewein, C., Kohl, R., and Brune, B. (2007) PPARgamma1 attenuates cytosol to membrane translocation of PKCalpha to desensitize monocytes/macrophages. *J. Cell Biol.* **176**, 681–694.
10. Ricote, M. and Glass, C.K. (2007) PPARs and molecular mechanisms of transrepression. *Biochim. Biophys. Acta* **1771**, 926–935.
11. Chen, F., Wang, M., O'Connor, J.P., He, M., Tripathi, T., and Harrison, L.E. (2003) Phosphorylation of PPARgamma via active ERK1/2 leads to its physical association with p65 and inhibition of NF-kappabeta. *J. Cell. Biochem.* **90**, 732–744.
12. Gupta, R.A., Polk, D.B., Krishna, U., Israel, D.A., Yan, F., DuBois, R.N., and Peek, R.M., Jr. (2001) Activation of peroxisome proliferator-activated receptor gamma suppresses nuclear factor kappa B-mediated apoptosis induced by Helicobacter pylori in gastric epithelial cells. *J. Biol. Chem.* **276**, 31059–31066.
13. Wang, P., Anderson, P.O., Chen, S., Paulsson, K.M., Sjogren, H.O., and Li, S. (2001) Inhibition of the transcription factors AP-1 and NF-kappaB in CD4 T cells by peroxisome proliferator-activated receptor gamma ligands. *Int. Immunopharmacol.* **1**, 803–812.
14. Yang, X.Y., Wang, L.H., Chen, T., Hodge, D.R., Resau, J.H., DaSilva, L., and Farrar, W.L. (2000) Activation of human T lymphocytes is inhibited by peroxisome proliferator-activated receptor gamma (PPARgamma) agonists. PPARgamma co-association with transcription factor NFAT. *J. Biol. Chem*. **275**, 4541–4544.
15. Subbaramaiah, K., Lin, D.T., Hart, J.C., and Dannenberg, A.J. (2001) Peroxisome proliferator-activated receptor gamma ligands suppress the transcriptional activation of cyclooxygenase-2. Evidence for involvement of activator protein-1 and CREB-binding protein/p300. *J. Biol. Chem*. **276**, 12440–12448.
16. Shipley, J.M. and Waxman, D.J. (2003) Down-regulation of STAT5b transcriptional activity by ligand-activated peroxisome proliferator-activated receptor (PPAR) alpha and PPARgamma. *Mol. Pharmacol.* **64**, 355–364.
17. Daynes, R.A. and Jones, D.C. (2002) Emerging roles of PPARs in inflammation and immunity. *Nat. Rev. Immunol.* **2**, 748–759.
18. Ramos, Y.F., Hestand, M.S., Verlaan, M., Krabbendam, E., Ariyurek, Y., van Galen, M., van Dam, H., van Ommen, G.J., den Dunnen, J.T., Zantema, A., and t Hoen, P.A. (2010) Genome-wide assessment of differential roles for p300 and CBP in transcription regulation. *Nucleic Acids Res*. **38**, 5396–5408.
19. Sahar, S., Reddy, M.A., Wong, C., Meng, L., Wang, M., and Natarajan, R. (2007) Cooperation of SRC-1 and p300 with NF-kappaB and CREB in angiotensin II-induced IL-6 expression in vascular smooth muscle cells. *Arterioscler. Thromb. Vasc. Biol.* **27**, 1528–1534.
20. Pascual, G., Fong, A.L., Ogawa, S., Gamliel, A., Li, A.C., Perissi, V., Rose, D.W., Willson, T.M., Rosenfeld, M.G., and Glass, C.K. (2005) A SUMOylation-dependent pathway mediates transrepression of inflammatory response genes by PPAR-gamma. *Nature* **437**, 759–763.
21. Jennewein, C., Kuhn, A.M., Schmidt, M.V., Meilladec-Jullig, V., von Knethen, A., Gonzalez, F.J., and Brune, B. (2008) Sumoylation of peroxisome proliferator-activated receptor gamma by apoptotic cells prevents lipopolysaccharide-induced NCoR removal from kappaB binding sites mediating transrepression of proinflammatory cytokines. *J. Immunol.* **181**, 5646–5652.
22. Park, J.Y., Bae, M.A., Cheon, H.G., Kim, S.S., Hong, J.M., Kim, T.H., Choi, J.Y., Kim, S.H., Lim, J., Choi, C.H., Shin, H.I., Kim, S.Y., and Park, E.K. (2009) A novel PPARgamma agonist, KR62776, suppresses RANKL-induced osteoclast differentiation and activity by inhibiting MAP kinase pathways. *Biochem. Biophys. Res. Commun.* **378**, 645–649.
23. Ji, H., Wang, H., Zhang, F., Li, X., Xiang, L., and Aiguo, S. (2010) PPARgamma agonist pioglitazone inhibits microglia inflammation by blocking p38 mitogen-activated protein kinase signaling pathways. *Inflamm. Res.* **59**, 921–929.

24. Desreumaux, P., Dubuquoy, L., Nutten, S., Peuchmaur, M., Englaro, W., Schoonjans, K., Derijard, B., Desvergne, B., Wahli, W., Chambon, P., Leibowitz, M.D., Colombel, J.F., and Auwerx, J. (2001) Attenuation of colon inflammation through activators of the retinoid X receptor (RXR)/peroxisome proliferator-activated receptor gamma (PPARgamma) heterodimer. A basis for new therapeutic strategies. *J. Exp. Med.* **193**, 827–838.
25. Johann, A.M., von Knethen, A., Lindemann, D., and Brune, B. (2006) Recognition of apoptotic cells by macrophages activates the peroxisome proliferator-activated receptor-gamma and attenuates the oxidative burst. *Cell Death Differ.* **13**, 1533–1540.
26. von Knethen, A., Tzieply, N., Jennewein, C., and Brune, B. (2010) Casein-kinase-II-dependent phosphorylation of PPARgamma provokes CRM1-mediated shuttling of PPARgamma from the nucleus to the cytosol. *J. Cell Sci.* **123**, 192–201.
27. Clark, R.B., Bishop-Bailey, D., Estrada-Hernandez, T., Hla, T., Puddington, L., and Padula, S.J. (2000) The nuclear receptor PPAR gamma and immunoregulation: PPAR gamma mediates inhibition of helper T cell responses. *J. Immunol.* **164**, 1364–1371.
28. Harris, S.G. and Phipps, R.P. (2001) The nuclear receptor PPAR gamma is expressed by mouse T lymphocytes and PPAR gamma agonists induce apoptosis. *Eur. J. Immunol.* **31**, 1098–1105.
29. Kim, E.J., Cho, D., Hwang, S.Y., and Kim, T.S. (2001) Interleukin-2 fusion protein with anti-CD3 single-chain Fv (sFv) selectively protects T cells from dexamethasone-induced apoptosis. *Vaccine* **20**, 608–615.
30. Cunard, R., Ricote, M., DiCampli, D., Archer, D.C., Kahn, D.A., Glass, C.K., and Kelly, C.J. (2002) Regulation of cytokine expression by ligands of peroxisome proliferator activated receptors. *J. Immunol.* **168**, 2795–2802.
31. Schmitz, J., Thiel, A., Kuhn, R., Rajewsky, K., Muller, W., Assenmacher, M., and Radbruch, A. (1994) Induction of interleukin 4 (IL-4) expression in T helper (Th) cells is not dependent on IL-4 from non-Th cells. *J. Exp. Med*. **179**, 1349–1353.
32. Yang, X.Y., Wang, L.H., Mihalic, K., Xiao, W., Chen, T., Li, P., Wahl, L.M., and Farrar, W.L. (2002) Interleukin (IL)-4 indirectly suppresses IL-2 production by human T lymphocytes via peroxisome proliferator-activated receptor gamma activated by macrophage-derived 12/15-lipoxygenase ligands. *J. Biol. Chem*. **277**, 3973–3978.
33. Chtanova, T., Kemp, R.A., Sutherland, A.P., Ronchese, F., and Mackay, C.R. (2001) Gene microarrays reveal extensive differential gene expression in both CD4(+) and CD8(+) type 1 and type 2 T cells. *J. Immunol.* **167**, 3057–3063.
34. Welch, J.S., Ricote, M., Akiyama, T.E., Gonzalez, F.J., and Glass, C.K. (2003) PPARgamma and PPARdelta negatively regulate specific subsets of lipopolysaccharide and IFN-gamma target genes in macrophages. *Proc. Natl. Acad. Sci. U. S. A.* **100**, 6712–6717.
35. Klotz, L., Burgdorf, S., Dani, I., Saijo, K., Flossdorf, J., Hucke, S., Alferink, J., Nowak, N., Beyer, M., Mayer, G., Langhans, B., Klockgether, T., Waisman, A., Eberl, G., Schultze, J., Famulok, M., Kolanus, W., Glass, C., Kurts, C., and Knolle, P.A. (2009) The nuclear receptor PPAR gamma selectively inhibits Th17 differentiation in a T cell-intrinsic fashion and suppresses CNS autoimmunity. *J. Exp. Med*. **206**, 2079–2089.
36. Chung, S.W., Kang, B.Y., Kim, S.H., Pak, Y.K., Cho, D., Trinchieri, G., and Kim, T.S. (2000) Oxidized low density lipoprotein inhibits interleukin-12 production in lipopolysaccharide-activated mouse macrophages via direct interactions between peroxisome proliferator-activated receptor-gamma and nuclear factor-kappa B. *J. Biol. Chem.* **275**, 32681–32687.
37. Zapolska-Downar, D. and Naruszewicz, M. (2009) Propionate reduces the cytokine-induced VCAM-1 and ICAM-1 expression by inhibiting nuclear factor-kappa B (NF-kappaB) activation. *J. Physiol. Pharmacol.* **60**, 123–131.
38. Lin, T.H., Yang, R.S., Tang, C.H., Lin, C.P., and Fu, W.M. (2007) PPARgamma inhibits osteogenesis via the down-regulation of the expression of COX-2 and iNOS in rats. *Bone* **41**, 562–574.
39. Liu, J.J., Liu, P.Q., Lin, D.J., Xiao, R.Z., Huang, M., Li, X.D., He, Y., and Huang, R.W. (2007) Downregulation of cyclooxygenase-2 expression and activation of caspase-3 are involved in peroxisome proliferator-activated receptor-gamma agonists induced apoptosis in human monocyte leukemia cells in vitro. *Ann. Hematol.* **86**, 173–183.
40. Kaplan, J.M., Cook, J.A., Hake, P.W., O'Connor, M., Burroughs, T.J., and Zingarelli, B. (2005) 15-Deoxy-delta(12,14)-prostaglandin J(2) (15D-PGJ(2)), a peroxisome proliferator activated receptor gamma ligand, reduces tissue leukosequestration and mortality in endotoxic shock. *Shock* **24**, 59–65.
41. Lee, K.J., Ha, E.S., Kim, M.K., Lee, S.H., Suh, J.S., Lee, S.H., Park, K.H., Park, J.H., Kim, D.J., Kang, D., Kim, B.C., Jeoung, D., Kim, Y.K., Kim, H.D., and Hahn, J.H. (2008) CD36 signaling inhibits the translation of heat shock protein 70 induced by oxidized low density lipoprotein through activation of peroxisome proliferators-activated receptor gamma. *Exp. Mol. Med*. **40**, 658–668.
42. Matsui, H., Asou, H., and Inaba, T. (2007) Cytokines direct the regulation of Bim mRNA stability by heat-shock cognate protein 70. *Mol. Cell* **25**, 99–112.
43. Kaplan, J., Cook, J.A., O'Connor, M., and Zingarelli, B. (2007) Peroxisome proliferator-activated receptor gamma is required for the inhibitory effect of ciglitazone but not 15-deoxy-delta 12,14-prostaglandin J2 on the NFkappaB pathway in human endothelial cells. *Shock* **28**, 722–726.
44. Straus, D.S., Pascual, G., Li, M., Welch, J.S., Ricote, M., Hsiang, C.H., Sengchanthalangsy, L.L., Ghosh, G., and Glass, C.K. (2000) 15-Deoxy-delta 12,14-prostaglandin J2 inhibits multiple steps in the NF-kappa B signaling pathway. *Proc. Natl. Acad. Sci. U. S. A.* **97**, 4844–4849.

45. Ayala, A., Wesche-Soldato, D.E., Perl, M., Lomas-Neira, J.L., Swan, R., and Chung, C.S. (2007) Blockade of apoptosis as a rational therapeutic strategy for the treatment of sepsis. *Novartis Found. Symp.* **280**, 37–49; discussion 49–52, 160–164.
46. Riedemann, N.C., Guo, R.F., and Ward, P.A. (2003) Novel strategies for the treatment of sepsis. *Nat. Med*. **9**, 517–524.
47. Hotchkiss, R.S., Chang, K.C., Swanson, P.E., Tinsley, K.W., Hui, J.J., Klender, P., Xanthoudakis, S., Roy, S., Black, C., Grimm, E., Aspiotis, R., Han, Y., Nicholson, D.W., and Karl, I.E. (2000) Caspase inhibitors improve survival in sepsis: a critical role of the lymphocyte. *Nat. Immunol.* **1**, 496–501.
48. Hotchkiss, R.S., Coopersmith, C.M., and Karl, I.E. (2005) Prevention of lymphocyte apoptosis--a potential treatment of sepsis? *Clin. Infect. Dis.* **41(Suppl 7)**, S465–469.
49. Hotchkiss, R.S., Coopersmith, C.M., McDunn, J.E., and Ferguson, T.A. (2009) The sepsis seesaw: tilting toward immunosuppression. *Nat. Med.* **15**, 496–497.
50. Hotchkiss, R.S., Tinsley, K.W., Swanson, P.E., Chang, K.C., Cobb, J.P., Buchman, T.G., Korsmeyer, S.J., and Karl, I.E. (1999) Prevention of lymphocyte cell death in sepsis improves survival in mice. *Proc. Natl. Acad. Sci. U. S. A.* **96**, 14541–14546.
51. Hotchkiss, R.S., Swanson, P.E., Knudson, C.M., Chang, K.C., Cobb, J.P., Osborne, D.F., Zollner, K.M., Buchman, T.G., Korsmeyer, S.J., and Karl, I.E. (1999) Overexpression of Bcl-2 in transgenic mice decreases apoptosis and improves survival in sepsis. *J. Immunol.* **162**, 4148–4156.
52. Peck-Palmer, O.M., Unsinger, J., Chang, K.C., McDonough, J.S., Perlman, H., McDunn, J.E., and Hotchkiss, R.S. (2009) Modulation of the Bcl-2 family blocks sepsis-induced depletion of dendritic cells and macrophages. *Shock* **31**, 359–366.
53. Wagner, T.H., Drewry, A.M., Macmillan, S., Dunne, W.M., Chang, K.C., Karl, I.E., Hotchkiss, R.S., and Cobb, J.P. (2007) Surviving sepsis: bcl-2 overexpression modulates splenocyte transcriptional responses in vivo. *Am. J. Physiol. Regul. Integr. Comp. Physiol.* **292**, R1751–1759.
54. Brahmamdam, P., Watanabe, E., Unsinger, J., Chang, K.C., Schierding, W., Hoekzema, A.S., Zhou, T.T., McDonough, J.S., Holemon, H., Heidel, J.D., Coopersmith, C.M., McDunn, J.E., and Hotchkiss, R.S. (2009) Targeted delivery of siRNA to cell death proteins in sepsis. *Shock* **32**, 131–139.
55. Hotchkiss, R.S., McConnell, K.W., Bullok, K., Davis, C.G., Chang, K.C., Schwulst, S.J., Dunne, J.C., Dietz, G.P., Bahr, M., McDunn, J.E., Karl, I.E., Wagner, T.H., Cobb, J.P., Coopersmith, C.M., and Piwnica-Worms, D. (2006) TAT-BH4 and TAT-Bcl-xL peptides protect against sepsis-induced lymphocyte apoptosis in vivo. *J. Immunol.* **176**, 5471–5477.
56. Schwulst, S.J., Muenzer, J.T., Peck-Palmer, O.M., Chang, K.C., Davis, C.G., McDonough, J.S., Osborne, D.F., Walton, A.H., Unsinger, J., McDunn, J.E., and Hotchkiss, R.S. (2008) Bim siRNA decreases lymphocyte apoptosis and improves survival in sepsis. *Shock* **30**, 127–134.
57. Strasser, A. and Pellegrini, M. (2004) T-lymphocyte death during shutdown of an immune response. *Trends Immunol.* **25**, 610–615.
58. Weber, S.U., Schewe, J.C., Lehmann, L.E., Muller, S., Book, M., Klaschik, S., Hoeft, A., and Stuber, F. (2008) Induction of Bim and Bid gene expression during accelerated apoptosis in severe sepsis. *Crit. Care* **12**, R128.
59. Soller, M., Tautenhahn, A., Brune, B., Zacharowski, K., John, S., Link, H., and von Knethen, A. (2006) Peroxisome proliferator-activated receptor gamma contributes to T lymphocyte apoptosis during sepsis. *J. Leukoc. Biol.* **79**, 235–243.
60. Tautenhahn, A., Brune, B., and von Knethen, A. (2003) Activation-induced PPARgamma expression sensitizes primary human T cells toward apoptosis. *J. Leukoc. Biol.* **73**, 665–672.
61. Patel, L., Pass, I., Coxon, P., Downes, C.P., Smith, S.A., and Macphee, C.H. (2001) Tumor suppressor and anti-inflammatory actions of PPARgamma agonists are mediated via upregulation of PTEN. *Curr. Biol.* **11**, 764–768.
62. Bonofiglio, D., Gabriele, S., Aquila, S., Catalano, S., Gentile, M., Middea, E., Giordano, F., and Ando, S. (2005) Estrogen receptor alpha binds to peroxisome proliferator-activated receptor response element and negatively interferes with peroxisome proliferator-activated receptor gamma signaling in breast cancer cells. *Clin. Cancer Res.* **11**, 6139–6147.
63. Teresi, R.E., Shaiu, C.W., Chen, C.S., Chatterjee, V.K., Waite, K.A., and Eng, C. (2006) Increased PTEN expression due to transcriptional activation of PPARgamma by lovastatin and rRosiglitazone. *Int. J. Cancer* **118**, 2390–2398.
64. Bonofiglio, D., Aquila, S., Catalano, S., Gabriele, S., Belmonte, M., Middea, E., Qi, H., Morelli, C., Gentile, M., Maggiolini, M., and Ando, S. (2006) Peroxisome proliferator-activated receptor-gamma activates p53 gene promoter binding to the nuclear factor-kappaB sequence in human MCF7 breast cancer cells. *Mol. Endocrinol.* **20**, 3083–3092.
65. Zaytseva, Y.Y., Wang, X., Southard, R.C., Wallis, N.K., and Kilgore, M.W. (2008) Down-regulation of PPARgamma1 suppresses cell growth and induces apoptosis in MCF-7 breast cancer cells. *Mol. Cancer* **7**, 90.
66. Wang, X., Southard, R.C., Allred, C.D., Talbert, D.R., Wilson, M.E., and Kilgore, M.W. (2008) MAZ drives tumor-specific expression of PPAR gamma 1 in breast cancer cells. *Breast Cancer Res. Treat.* **111**, 103–111.
67. Wang, X., Southard, R.C., and Kilgore, M.W. (2004) The increased expression of peroxisome proliferator-activated receptor-gamma1 in human breast cancer is mediated by selective promoter usage. *Cancer Res.* **64**, 5592–5596.

68. Yu, J., Shen, B., Chu, E.S., Teoh, N., Cheung, K.F., Wu, C.W., Wang, S., Lam, C.N., Feng, H., Zhao, J., Cheng, A.S., To, K.F., Chan, H.L., and Sung, J.J. (2010) Inhibitory role of peroxisome proliferator-activated receptor gamma in hepatocarcinogenesis in mice and in vitro. *Hepatology* **51**, 2008–2019.
69. Baek, S.J., Kim, J.S., Jackson, F.R., Eling, T.E., McEntee, M.F., and Lee, S.H. (2004) Epicatechin gallate-induced expression of NAG-1 is associated with growth inhibition and apoptosis in colon cancer cells. *Carcinogenesis* **25**, 2425–2432.
70. Chen, Y.L., Lin, P.C., Chen, S.P., Lin, C.C., Tsai, N.M., Cheng, Y.L., Chang, W.L., Lin, S.Z., and Harn, H.J. (2007) Activation of nonsteroidal anti-inflammatory drug-activated gene-1 via extracellular signal-regulated kinase 1/2 mitogen-activated protein kinase revealed a isochaihulactone-triggered apoptotic pathway in human lung cancer A549 cells. *J. Pharmacol. Exp. Ther.* **323**, 746–756.
71. Liu, T., Bauskin, A.R., Zaunders, J., Brown, D.A., Pankhurst, S., Russell, P.J., and Breit, S.N. (2003) Macrophage inhibitory cytokine 1 reduces cell adhesion and induces apoptosis in prostate cancer cells. *Cancer Res*. **63**, 5034–5040.
72. Kroll, T.G., Sarraf, P., Pecciarini, L., Chen, C.J., Mueller, E., Spiegelman, B.M., and Fletcher, J.A. (2000) PAX8-PPARgamma1 fusion oncogene in human thyroid carcinoma [corrected]. *Science* **289**, 1357–1360.
73. Eberhardt, N.L., Grebe, S.K., McIver, B., and Reddi, H.V. (2010) The role of the PAX8/PPARgamma fusion oncogene in the pathogenesis of follicular thyroid cancer. *Mol. Cell. Endocrinol.* **321**, 50–56.
74. Lui, W.O., Foukakis, T., Liden, J., Thoppe, S.R., Dwight, T., Hoog, A., Zedenius, J., Wallin, G., Reimers, M., and Larsson, C. (2005) Expression profiling reveals a distinct transcription signature in follicular thyroid carcinomas with a PAX8-PPAR(gamma) fusion oncogene. *Oncogene* **24**, 1467–1476.
75. Inamoto, T., Shah, J.B., and Kamat, A.M. (2009) Friend or foe? Role of peroxisome proliferator-activated receptor-gamma in human bladder cancer. *Urol. Oncol.* **27**, 585–591.
76. Kang, D.W., Choi, C.H., Park, J.Y., Kang, S.K., and Kim, Y.K. (2008) Ciglitazone induces caspase-independent apoptosis through down-regulation of XIAP and survivin in human glioma cells. *Neurochem. Res.* **33**, 551–561.
77. Dranoff, G. (2004) Cytokines in cancer pathogenesis and cancer therapy. *Nat. Rev. Cancer* **4**, 11–22.
78. Lin, W.W. and Karin, M. (2007) A cytokine-mediated link between innate immunity, inflammation, and cancer. *J. Clin. Invest*. **117**, 1175–1183.
79. Michalik, L. and Wahli, W. (2008) PPARs mediate lipid signaling in inflammation and cancer. *PPAR Res*. **2008**, 134059.
80. Karin, M. (2008) The IkappaB kinase - a bridge between inflammation and cancer. *Cell Res.* **18**, 334–342.
81. Van Ginderachter, J.A., Movahedi, K., Van den Bossche, J., and De Baetselier, P. (2008) Macrophages, PPARs, and cancer. *PPAR Res*. **2008**, 169414.
82. Solinas, G., Germano, G., Mantovani, A., and Allavena, P. (2009) Tumor-associated macrophages (TAM) as major players of the cancer-related inflammation. *J. Leukoc. Biol.* **86**, 1065–1073.
83. Fauconnet, S., Lascombe, I., Chabannes, E., Adessi, G.L., Desvergne, B., Wahli, W., and Bittard, H. (2002) Differential regulation of vascular endothelial growth factor expression by peroxisome proliferator-activated receptors in bladder cancer cells. *J. Biol. Chem.* **277**, 23534–23543.
84. Bouhlel, M.A., Derudas, B., Rigamonti, E., Dievart, R., Brozek, J., Haulon, S., Zawadzki, C., Jude, B., Torpier, G., Marx, N., Staels, B., and Chinetti-Gbaguidi, G. (2007) PPARgamma activation primes human monocytes into alternative M2 macrophages with anti-inflammatory properties. *Cell Metab.* **6**, 137–143.
85. Odegaard, J.I., Ricardo-Gonzalez, R.R., Goforth, M.H., Morel, C.R., Subramanian, V., Mukundan, L., Red Eagle, A., Vats, D., Brombacher, F., Ferrante, A.W., and Chawla, A. (2007) Macrophage-specific PPARgamma controls alternative activation and improves insulin resistance. *Nature* **447**, 1116–1120.
86. Olefsky, J.M. and Glass, C.K. (2010) Macrophages, inflammation, and insulin resistance. *Annu. Rev. Physiol.* **72**, 219–246.
87. Van Ginderachter, J.A., Meerschaut, S., Liu, Y., Brys, L., De Groeve, K., Hassanzadeh Ghassabeh, G., Raes, G., and De Baetselier, P. (2006) Peroxisome proliferator-activated receptor gamma (PPARgamma) ligands reverse CTL suppression by alternatively activated (M2) macrophages in cancer. *Blood* **108**, 525–535.
88. Shah, Y.M., Morimura, K., and Gonzalez, F.J. (2007) Expression of peroxisome proliferator-activated receptor-gamma in macrophage suppresses experimentally induced colitis. *Am. J. Physiol. Gastrointest. Liver Physiol.* **292**, G657–666.
89. Wei, S., Kulp, S.K., and Chen, C.S. (2010) Energy restriction as an antitumor target of thiazolidinediones. *J. Biol. Chem.* **285**, 9780–9791.
90. Galli, A., Ceni, E., Mello, T., Polvani, S., Tarocchi, M., Buccoliero, F., Lisi, F., Cioni, L., Ottanelli, B., Foresta, V., Mastrobuoni, G., Moneti, G., Pieraccini, G., Surrenti, C., and Milani, S. (2010) Thiazolidinediones inhibit hepatocarcinogenesis in hepatitis B virus-transgenic mice by peroxisome proliferator-activated receptor gamma-independent regulation of nucleophosmin. *Hepatology* **52**, 493–505.
91. Galli, A., Ceni, E., Crabb, D.W., Mello, T., Salzano, R., Grappone, C., Milani, S., Surrenti, E., Surrenti, C., and Casini, A. (2004) Antidiabetic thiazolidinediones inhibit invasiveness of pancreatic cancer cells via PPARgamma independent mechanisms. *Gut* **53**, 1688–1697.

92. Ricote, M., Valledor, A.F., and Glass, C.K. (2004) Decoding transcriptional programs regulated by PPARs and LXRs in the macrophage: effects on lipid homeostasis, inflammation, and atherosclerosis. *Arterioscler. Thromb. Vasc. Biol.* **24**, 230–239.
93. Roberts, A.W., Thomas, A., Rees, A., and Evans, M. (2003) Peroxisome proliferator-activated receptor-gamma agonists in atherosclerosis: current evidence and future directions. *Curr. Opin. Lipidol.* **14**, 567–573.
94. Yki-Jarvinen, H. (2004) Thiazolidinediones. *N. Engl. J. Med.* **351**, 1106–1118.
95. Wang, M. and Tafuri, S. (2003) Modulation of PPARgamma activity with pharmaceutical agents: treatment of insulin resistance and atherosclerosis. *J. Cell Biochem.* **89**, 38–47.
96. Kaul, S., Bolger, A.F., Herrington, D., Giugliano, R.P., and Eckel, R.H. (2010) Thiazolidinedione drugs and cardiovascular risks: a science advisory from the American Heart Association and American College of Cardiology Foundation. *Circulation* **121**, 1868–1877.
97. Langenfeld, M.R., Forst, T., Hohberg, C., Kann, P., Lubben, G., Konrad, T., Fullert, S.D., Sachara, C., and Pfutzner, A. (2005) Pioglitazone decreases carotid intima-media thickness independently of glycemic control in patients with type 2 diabetes mellitus: results from a controlled randomized study. *Circulation* **111**, 2525–2531.
98. Li, D., Chen, K., Sinha, N., Zhang, X., Wang, Y., Sinha, A.K., Romeo, F., and Mehta, J.L. (2005) The effects of PPAR-gamma ligand pioglitazone on platelet aggregation and arterial thrombus formation. *Cardiovasc. Res*. **65**, 907–912.
99. Derosa, G., Cicero, A.F., Gaddi, A., Ragonesi, P.D., Piccinni, M.N., Fogari, E., Salvadeo, S., Ciccarelli, L., and Fogari, R. (2005) A comparison of the effects of pioglitazone and rosiglitazone combined with glimepiride on prothrombotic state in type 2 diabetic patients with the metabolic syndrome. *Diabetes Res. Clin. Pract.* **69**, 5–13.
100. Marx, N., Walcher, D., Ivanova, N., Rautzenberg, K., Jung, A., Friedl, R., Hombach, V., de Caterina, R., Basta, G., Wautier, M.P., and Wautiers, J.L. (2004) Thiazolidinediones reduce endothelial expression of receptors for advanced glycation end products. *Diabetes* **53**, 2662–2668.
101. Majuri, A., Santaniemi, M., Rautio, K., Kunnari, A., Vartiainen, J., Ruokonen, A., Kesaniemi, Y.A., Tapanainen, J.S., Ukkola, O., and Morin-Papunen, L. (2007) Rosiglitazone treatment increases plasma levels of adiponectin and decreases levels of resistin in overweight women with PCOS: a randomized placebo-controlled study. *Eur. J. Endocrinol.* **156**, 263–269.
102. Wang, T.D., Chen, W.J., Cheng, W.C., Lin, J.W., Chen, M.F., and Lee, Y.T. (2006) Relation of improvement in endothelium-dependent flow-mediated vasodilation after rosiglitazone to changes in asymmetric dimethylarginine, endothelin-1, and C-reactive protein in nondiabetic patients with the metabolic syndrome. *Am. J. Cardiol.* **98**, 1057–1062.
103. Szapary, P.O., Bloedon, L.T., Samaha, F.F., Duffy, D., Wolfe, M.L., Soffer, D., Reilly, M.P., Chittams, J., and Rader, D.J. (2006) Effects of pioglitazone on lipoproteins, inflammatory markers, and adipokines in nondiabetic patients with metabolic syndrome. *Arterioscler. Thromb. Vasc. Biol.* **26**, 182–188.
104. Haffner, S.M., Greenberg, A.S., Weston, W.M., Chen, H., Williams, K., and Freed, M.I. (2002) Effect of rosiglitazone treatment on nontraditional markers of cardiovascular disease in patients with type 2 diabetes mellitus. *Circulation* **106**, 679–684.
105. Ross, R. (1999) Atherosclerosis--an inflammatory disease. *N. Engl. J. Med.* **340**, 115–126.
106. Collins, A.R., Meehan, W.P., Kintscher, U., Jackson, S., Wakino, S., Noh, G., Palinski, W., Hsueh, W.A., and Law, R.E. (2001) Troglitazone inhibits formation of early atherosclerotic lesions in diabetic and nondiabetic low density lipoprotein receptor-deficient mice. *Arterioscler. Thromb. Vasc. Biol.* **21**, 365–371.
107. Babaev, V.R., Yancey, P.G., Ryzhov, S.V., Kon, V., Breyer, M.D., Magnuson, M.A., Fazio, S., and Linton, M.F. (2005) Conditional knockout of macrophage PPARgamma increases atherosclerosis in C57BL/6 and low-density lipoprotein receptor-deficient mice. *Arterioscler. Thromb. Vasc. Biol.* **25**, 1647–1653.
108. Woollard, K.J. and Geissmann, F. (2010) Monocytes in atherosclerosis: subsets and functions. *Nat. Rev. Cardiol.* **7**, 77–86.
109. Galkina, E. and Ley, K. (2009) Immune and inflammatory mechanisms of atherosclerosis (*). *Annu. Rev. Immunol.* **27**, 165–197.
110. Moore, K.J., Rosen, E.D., Fitzgerald, M.L., Randow, F., Andersson, L.P., Altshuler, D., Milstone, D.S., Mortensen, R.M., Spiegelman, B.M., and Freeman, M.W. (2001) The role of PPAR-gamma in macrophage differentiation and cholesterol uptake. *Nat. Med.* **7**, 41–47.
111. Chinetti, G., Lestavel, S., Bocher, V., Remaley, A.T., Neve, B., Torra, I.P., Teissier, E., Minnich, A., Jaye, M., Duverger, N., Brewer, H.B., Fruchart, J.C., Clavey, V., and Staels, B. (2001) PPAR-alpha and PPAR-gamma activators induce cholesterol removal from human macrophage foam cells through stimulation of the ABCA1 pathway. *Nat. Med.* **7**, 53–58.
112. Akiyama, T.E., Sakai, S., Lambert, G., Nicol, C.J., Matsusue, K., Pimprale, S., Lee, Y.H., Ricote, M., Glass, C.K., Brewer, H.B., Jr., and Gonzalez, F.J. (2002) Conditional disruption of the peroxisome proliferator-activated receptor gamma gene in mice results in lowered expression of ABCA1, ABCG1, and apoE in macrophages and reduced cholesterol efflux. *Mol. Cell. Biol*. **22**, 2607–2619.
113. Chawla, A., Barak, Y., Nagy, L., Liao, D., Tontonoz, P., and Evans, R.M. (2001) PPAR-gamma dependent and independent effects on macrophage-gene expression in lipid metabolism and inflammation. *Nat. Med*. **7**, 48–52.

114. Mosser, D.M. and Edwards, J.P. (2008) Exploring the full spectrum of macrophage activation. *Nat. Rev. Immunol.* **8**, 958–969.
115. Gordon, S. (2003) Alternative activation of macrophages. *Nat. Rev. Immunol.* **3**, 23–35.
116. Edwards, J.P., Zhang, X., Frauwirth, K.A., and Mosser, D.M. (2006) Biochemical and functional characterization of three activated macrophage populations. *J. Leukoc. Biol.* **80**, 1298–1307.
117. Mackaness, G.B. (1964) The immunological basis of acquired cellular resistance. *J. Exp. Med.* **120**, 105–120.
118. Murray, H.W. (2008) Accelerated control of visceral Leishmania donovani infection in interleukin-6-deficient mice. *Infect. Immun.* **76**, 4088–4091.
119. Jennewein, C., von Knethen, A., Schmid, T., and Brune, B. (2010) MicroRNA-27b contributes to lipopolysaccharide-mediated peroxisome proliferator-activated receptor gamma (PPARgamma) mRNA destabilization. *J. Biol. Chem*. **285**, 11846–11853.
120. Necela, B.M., Su, W., and Thompson, E.A. (2008) Toll-like receptor 4 mediates cross-talk between peroxisome proliferator-activated receptor gamma and nuclear factor-kappaB in macrophages. *Immunology* **125**, 344–358.
121. Zhou, M., Wu, R., Dong, W., Jacob, A., and Wang, P. (2008) Endotoxin downregulates peroxisome proliferator-activated receptor-gamma via the increase in TNF-alpha release. *Am. J. Physiol. Regul. Integr. Comp. Physiol.* **294**, R84–92.
122. Mendes Sdos, S., Candi, A., Vansteenbrugge, M., Pignon, M.R., Bult, H., Boudjeltia, K.Z., Munaut, C., and Raes, M. (2009) Microarray analyses of the effects of NF-kappaB or PI3K pathway inhibitors on the LPS-induced gene expression profile in RAW264.7 cells: synergistic effects of rapamycin on LPS-induced MMP9-overexpression. *Cell. Signal.* **21**, 1109–1122.
123. von Knethen, A.A. and Brune, B. (2001) Delayed activation of PPARgamma by LPS and IFN-gamma attenuates the oxidative burst in macrophages. *FASEB J.* **15**, 535–544.
124. Mantovani, A., Allavena, P., Sozzani, S., Vecchi, A., Locati, M., and Sica, A. (2004) Chemokines in the recruitment and shaping of the leukocyte infiltrate of tumors. *Semin. Cancer Biol.* **14**, 155–160.
125. Porcheray, F., Viaud, S., Rimaniol, A.C., Leone, C., Samah, B., Dereuddre-Bosquet, N., Dormont, D., and Gras, G. (2005) Macrophage activation switching: an asset for the resolution of inflammation. *Clin. Exp. Immunol.* **142**, 481–489.
126. Li, A.C., Brown, K.K., Silvestre, M.J., Willson, T.M., Palinski, W., and Glass, C.K. (2000) Peroxisome proliferator-activated receptor gamma ligands inhibit development of atherosclerosis in LDL receptor-deficient mice. *J. Clin. Invest.* **106**, 523–531.
127. Chen, Z., Ishibashi, S., Perrey, S., Osuga, J., Gotoda, T., Kitamine, T., Tamura, Y., Okazaki, H., Yahagi, N., Iizuka, Y., Shionoiri, F., Ohashi, K., Harada, K., Shimano, H., Nagai, R., and Yamada, N. (2001) Troglitazone inhibits atherosclerosis in apolipoprotein E-knockout mice: pleiotropic effects on CD36 expression and HDL. *Arterioscler. Thromb. Vasc. Biol*. **21**, 372–377.
128. Nakaya, H., Summers, B.D., Nicholson, A.C., Gotto, A.M., Jr., Hajjar, D.P., and Han, J. (2009) Atherosclerosis in LDLR-knockout mice is inhibited, but not reversed, by the PPARgamma ligand pioglitazone. *Am. J. Pathol.* **174**, 2007–2014.
129. Thorp, E., Kuriakose, G., Shah, Y.M., Gonzalez, F.J., and Tabas, I. (2007) Pioglitazone increases macrophage apoptosis and plaque necrosis in advanced atherosclerotic lesions of nondiabetic low-density lipoprotein receptor-null mice. *Circulation* **116**, 2182–2190.
130. Hu, Q., Zhang, X.J., Zhang, C., Zhao, Y.X., He, H., Liu, C.X., Feng, J.B., Jiang, H., Yang, F.L., Zhang, C.X., and Zhang, Y. (2008) Peroxisome proliferator-activated receptor-gamma1 gene therapy attenuates atherosclerosis and stabilizes plaques in apolipoprotein E-deficient mice. *Hum. Gene Ther.* **19**, 287–299.
131. Tabas, I. (2010) Macrophage death and defective inflammation resolution in atherosclerosis. *Nat. Rev. Immunol.* **10**, 36–46.
132. Weber, C., Zernecke, A., and Libby, P. (2008) The multifaceted contributions of leukocyte subsets to atherosclerosis: lessons from mouse models. *Nat. Rev. Immunol.* **8**, 802–815.
133. Natarajan, C., Muthian, G., Barak, Y., Evans, R.M., and Bright, J.J. (2003) Peroxisome proliferator-activated receptor-gamma-deficient heterozygous mice develop an exacerbated neural antigen-induced Th1 response and experimental allergic encephalomyelitis. *J. Immunol.* **171**, 5743–5750.
134. Feinstein, D.L., Galea, E., Gavrilyuk, V., Brosnan, C.F., Whitacre, C.C., Dumitrescu-Ozimek, L., Landreth, G.E., Pershadsingh, H.A., Weinberg, G., and Heneka, M.T. (2002) Peroxisome proliferator-activated receptor-gamma agonists prevent experimental autoimmune encephalomyelitis. *Ann. Neurol.* **51**, 694–702.
135. Kielian, T. and Drew, P.D. (2003) Effects of peroxisome proliferator-activated receptor-gamma agonists on central nervous system inflammation. *J. Neurosci. Res.* **71**, 315–325.
136. Niino, M., Iwabuchi, K., Kikuchi, S., Ato, M., Morohashi, T., Ogata, A., Tashiro, K., and Onoe, K. (2001) Amelioration of experimental autoimmune encephalomyelitis in C57BL/6 mice by an agonist of peroxisome proliferator-activated receptor-gamma. *J. Neuroimmunol.* **116**, 40–48.
137. Su, C.G., Wen, X., Bailey, S.T., Jiang, W., Rangwala, S.M., Keilbaugh, S.A., Flanigan, A., Murthy, S., Lazar, M.A., and Wu, G.D. (1999) A novel therapy for colitis utilizing PPAR-gamma ligands to inhibit the epithelial inflammatory response. *J. Clin. Invest.* **104**, 383–389.

138. Sanchez-Hidalgo, M., Martin, A.R., Villegas, I., and de la Lastra, C.A. (2007) Rosiglitazone, a PPARgamma ligand, modulates signal transduction pathways during the development of acute TNBS-induced colitis in rats. *Eur. J. Pharmacol.* **562**, 247–258.
139. Tanaka, T., Kohno, H., Yoshitani, S., Takashima, S., Okumura, A., Murakami, A., and Hosokawa, M. (2001) Ligands for peroxisome proliferator-activated receptors alpha and gamma inhibit chemically induced colitis and formation of aberrant crypt foci in rats. *Cancer Res.* **61**, 2424–2428.
140. Saubermann, L.J., Nakajima, A., Wada, K., Zhao, S., Terauchi, Y., Kadowaki, T., Aburatani, H., Matsuhashi, N., Nagai, R., and Blumberg, R.S. (2002) Peroxisome proliferator-activated receptor gamma agonist ligands stimulate a Th2 cytokine response and prevent acute colitis. *Inflamm. Bowel Dis.* **8**, 330–339.
141. Cuzzocrea, S., Mazzon, E., Dugo, L., Patel, N.S., Serraino, I., Di Paola, R., Genovese, T., Britti, D., De Maio, M., Caputi, A.P., and Thiemermann, C. (2003) Reduction in the evolution of murine type II collagen-induced arthritis by treatment with rosiglitazone, a ligand of the peroxisome proliferator-activated receptor gamma. *Arthritis Rheum.* **48**, 3544–3556.
142. Setoguchi, K., Misaki, Y., Terauchi, Y., Yamauchi, T., Kawahata, K., Kadowaki, T., and Yamamoto, K. (2001) Peroxisome proliferator-activated receptor-gamma haploinsufficiency enhances B cell proliferative responses and exacerbates experimentally induced arthritis. *J. Clin. Invest.* **108**, 1667–1675.
143. Shiojiri, T., Wada, K., Nakajima, A., Katayama, K., Shibuya, A., Kudo, C., Kadowaki, T., Mayumi, T., Yura, Y., and Kamisaki, Y. (2002) PPAR gamma ligands inhibit nitrotyrosine formation and inflammatory mediator expressions in adjuvant-induced rheumatoid arthritis mice. *Eur. J. Pharmacol.* **448**, 231–238.
144. Xu, H., Barnes, G.T., Yang, Q., Tan, G., Yang, D., Chou, C.J., Sole, J., Nichols, A., Ross, J.S., Tartaglia, L.A., and Chen, H. (2003) Chronic inflammation in fat plays a crucial role in the development of obesity-related insulin resistance. *J. Clin. Invest.* **112**, 1821–1830.
145. Lee, K.S., Park, S.J., Kim, S.R., Min, K.H., Jin, S.M., Lee, H.K., and Lee, Y.C. (2006) Modulation of airway remodeling and airway inflammation by peroxisome proliferator-activated receptor gamma in a murine model of toluene diisocyanate-induced asthma. *J. Immunol.* **177**, 5248–5257.
146. Belvisi, M.G., Hele, D.J., and Birrell, M.A. (2006) Peroxisome proliferator-activated receptor gamma agonists as therapy for chronic airway inflammation. *Eur. J. Pharmacol.* **533**, 101–109.
147. Ivashchenko, C.Y., Duan, S.Z., Usher, M.G., and Mortensen, R.M. (2007) PPAR-gamma knockout in pancreatic epithelial cells abolishes the inhibitory effect of rosiglitazone on caerulein-induced acute pancreatitis. *Am. J. Physiol. Gastrointest. Liver Physiol.* **293**, G319–326.
148. Demerjian, M., Man, M.Q., Choi, E.H., Brown, B.E., Crumrine, D., Chang, S., Mauro, T., Elias, P.M., and Feingold, K.R. (2006) Topical treatment with thiazolidinediones, activators of peroxisome proliferator-activated receptor-gamma, normalizes epidermal homeostasis in a murine hyperproliferative disease model. *Exp. Dermatol.* **15**, 154–160.
149. Cuzzocrea, S., Pisano, B., Dugo, L., Ianaro, A., Maffia, P., Patel, N.S., Di Paola, R., Ialenti, A., Genovese, T., Chatterjee, P.K., Di Rosa, M., Caputi, A.P., and Thiemermann, C. (2004) Rosiglitazone, a ligand of the peroxisome proliferator-activated receptor-gamma, reduces acute inflammation. *Eur. J. Pharmacol.* **483**, 79–93.
150. Hall, J.M. and McDonnell, D.P. (2007) The molecular mechanisms underlying the proinflammatory actions of thiazolidinediones in human macrophages. *Mol. Endocrinol.* **21**, 1756–1768.
